# Trajectories of kidney function over 10 years in patients with chronic kidney disease: a 10 year follow-up of FROM-J study

**DOI:** 10.1007/s10157-026-02820-1

**Published:** 2026-01-31

**Authors:** Reiko Okubo, Masahide Kondo, Chie Saito, Hirayasu Kai, Ryoya Tsunoda, Akihiko Kato, Shoichi Maruyama, Jun Wada, Takashi Wada, Ichiei Narita, Kunihiro Yamagata

**Affiliations:** 1 Department of Nephrology, Institute of Medicine, University of Tsukuba, 1-1-1 Ten-Oudai, Tsukuba, Ibaraki Japan; 2 Department of Health Care Policy and Health Economics, Institute of Medicine, University of Tsukuba, Ibaraki, Japan; 3 Department of Clinical Laboratory Medicine, Institute of Medicine, University of Tsukuba, Ibaraki, Japan; 4 Ibaraki Clinical Education and Training Center, Institute of Medicine, University of Tsukuba, Tsukuba, Ibaraki Japan; 5 Department of Nephrology, Kosai Municipal Hospital, Kosai, Shizuoka Japan; 6 Department of Nephrology, Nagoya University Graduate School of Medicine, Nagoya, Japan; 7 Department of Nephrology, Rheumatology, Endocrinology and Metabolism, Okayama University Graduate School of Medicine, Dentistry and Pharmaceutical Sciences, Okayama, Japan; 8 Department of Nephrology and Rheumatology, Kanazawa University, Kanazawa, Japan; 9 Niigata Institute for Health and Sports Medicine, Niigata, Japan

**Keywords:** Chronic kidney disease (CKD), Kidney function, Estimated glomerular filtration rate (eGFR), Trajectory, Group-based trajectory modeling (GBTM)

## Abstract

**Background:**

The frontier of renal outcome modifications in Japan 10 (FROM-J10) study is a 10 year longitudinal cohort study evaluating the long-term outcomes of treatment according to the clinical guidelines for chronic kidney disease (CKD) by primary care physicians. This study aimed to identify distinctive trajectories of kidney function among patients with CKD and evaluate the patient characteristics associated with each trajectory using the FROM-J10 study data.

**Method:**

This secondary study used 10 years of data from 2379 patients aged between 40 and 74 years with CKD stages from G1 to G5 in the FROM-J10 study. Group-based trajectory modeling was applied to the change in estimated glomerular filtration rate (eGFR) over time, and patients were classified into distinct groups that followed similar trajectories. Multivariate logistic analysis was performed for patient characteristics associated with each trajectory.

**Results:**

In total, 2257 patients with at least three eGFR values were included in this study. Two distinct trajectories of eGFR decline were identified: progressive decline (*n* = 1240, 54.9%) and gradual decline (*n* = 1017, 45.1%). In multivariate logistic analysis with gradual eGFR decline as a reference, proteinuria was associated with progressive eGFR decline in CKD from G2 to G4 + 5; lower albumin in G2, G3a, and G4 + 5; and lower hemoglobin in G3a to G4 + 5.

**Conclusions:**

In patients with CKD adequately treated by primary care physicians, kidney function declined very slowly over 10 years. We suggest that patient characteristics identified as progressive eGFR decline, proteinuria, and lower albumin and hemoglobin levels should be managed appropriately in clinical practice.

**Supplementary Information:**

The online version contains supplementary material available at 10.1007/s10157-026-02820-1.

## Introduction

Kidney function declines naturally with age and takes different courses depending on individual characteristics [1, 2]. The estimated glomerular filtration rate (eGFR) is an important clinical indicator of changes in kidney function, and various approaches have been applied to measure its longitudinal changes [3, 4]. Most studies have used analytical approaches that assumed the linearity of changes in eGFR, including the use of absolute change in eGFR, percentage change in eGFR over time, and change in eGFR category or stage [1, 5–8]. However, nonlinear changes in kidney function have been reported in cases with long observation periods, high baseline eGFR, or immediately before end-stage renal disease [9–13].

Group-based trajectory modeling (GBTM) is a statistical method designed to identify distinct subgroups of patients who share similar longitudinal trajectories of disease progression or clinical outcomes. By utilizing longitudinal data, GBTM enables researchers to capture complex and heterogeneous temporal patterns that may be obscured in conventional analytical approaches [14, 15]. This method has been applied in clinical and epidemiological studies involving chronic diseases [9, 11, 13] because it flexibly accommodates nonlinear changes and missing data. O’Hare et al. [9] described the eGFR trajectories of more than 5000 United States veterans during a 2 year period before dialysis initiation. They identified 4 distinct trajectories: persistent low eGFR, progressive eGFR loss, accelerated eGFR loss, and catastrophic eGFR loss. They found that patients with more rapid loss of eGFR trajectories were more likely to have been hospitalized and have an inpatient diagnosis of acute kidney injury and were less likely to have received pre-dialysis care.

The Frontier of Renal Outcome Modifications in Japan 10 (FROM-J10) study is a 10 year follow-up study of patients with chronic kidney disease (CKD) enrolled in the FROM-J study, a cluster-randomized controlled study that evaluated the effectiveness of CKD clinical guideline-based treatment [16] by primary care physicians in cooperation with nephrologists [7, 8]. From April 1, 2008 to October 19, 2008, 2379 patients with CKD aged between 40 and 74 years were enrolled from 49 local medical associations and 15 different prefectures in the FROM-J study. A total of 1473 participants (61.9%) had continued clinical visits to primary care physicians and up to 17 eGFR values were measured over 10 years. Imasawa et al. [8] calculated the rate of eGFR decline using a generalized linear model or mixed model based on eGFR values in the FROM-J10 study. They reported that kidney function declined at all CKD stages over 10 years, with an overall rate at −2.27 ± 3.97 mL/min/1.73 m^2^/year and the slowest rate at −2.00 ± 3.45 mL/min/1.73 m^2^/year in CKD stage G3a. The FROM-J10 study, longitudinal cohort studies targeting patients over 10 years, is extremely valuable. There are several cohort studies targeting patients with CKD over 3 to 4 years [5–7] or studies using annual health examination data from the general population [1, 13]. Secondary research using FROM-J10 which measured quality of life scores in long-term CKD survivors has recently been reported [17]. Therefore, we aimed to identify distinctive trajectories of kidney function among patients with CKD using the GBTM and evaluate patient characteristics associated with each trajectory using 10 years of data from the FROM-J10 study.

## Methods

### Study design

This was a secondary study using 10 years of data from patients with CKD enrolled in the FROM-J study from April 1, 2008 to October 19, 2008, and followed for 10 years in the FROM-J10 study until March 31, 2019. Data collection was performed by the data center every 6 months for an intervention period of 3.5 years. During the observation period after 3.5 years, data collection was performed by either primary care physicians or patients filling out the questionnaires at the 5th and 10th years. Detailed designs and main results have been reported previously [7, 8]. This study was conducted in accordance with the 1964 Declaration of Helsinki and its later amendments and was approved by the ethics committee of clinical research at the University of Tsukuba (No. 80 and 1311).

### Participants

In the FROM-J study, included patients were aged between 40 and 74 years in CKD stage G1, G2, G4 or G5, or CKD stage G3a/G3b with proteinuria (ratio of urinary protein/urinary creatinine ≥ 0.3 g/g, creatinine, or proteinuria ≥ 1 +) and diabetes or hypertension, who were under consultation with primary care physicians. Patients undergoing dialysis or renal transplantation were excluded. For the initial 3.5 years of the intervention study, participants were assigned to two intervention groups as follows: standard intervention, standard treatment according to CKD clinical guidelines [16]; and advanced intervention based on a practice facilitation program. The practice facilitation program comprised patient education by dieticians at primary care physicians’ clinics, newsletters to patients, patient encouragement to visit PCPs during interruptions, alerts to PCPs to inform the timing of referral to nephrologists, and support for PCPs to control patient data [7].

### Study variables

Baseline demographic and clinical characteristics, laboratory data (serum albumin, hemoglobin, and serum creatinine), and presence of comorbidities, hypertension, diabetes, hyperlipidemia, and hyperuricemia, were based on the FROM-J study. The eGFR was calculated from serum creatine, age, and sex using the Japanese equation as follows: eGFR (mL/min/1.73 m^2^) = 194 × serum creatinine^−1.094^ × age^−0.287^ × 0.739 (for female). eGFR data were collected every 6 months and up to 16 measurements were taken during the observation period.

### Statistical analysis

Statistical analysis consisted of three consecutive steps: (1) determining the trajectories of eGFR changes during the observation period, (2) classifying patient characteristics and estimating the eGFR slope for each identified trajectory and CKD stage, and (3) assessing patient characteristics associated with each identified trajectory.

For the first step, we identified patterns of eGFR change over 10 years (April 2008 to October 2018) using a GBTM implemented in Stata SE software version 18 (StataCorp LLC, College Station, TX, USA). The GBTM is a statistical application of finite mixture modeling that aims to assign individual patients to distinct subgroups that follow similar trajectories (patterns of change) by modeling between-person differences in within-person changes on longitudinal observational data. To select an appropriate model, we performed the analysis with reference to the proposed frameworks [14, 15]. We sequentially generated models in which the number of trajectories ranged from two to three, with reference to past studies [8, 11, 13]. The shapes of the trajectories were represented by polynomial functions ranging from the first to third order (linear, quadratic, or cubic). The appropriate model was determined using the following adequacy criteria: (a) the average posterior probability of assignments for each trajectory should be 0.7; (b) the odds of correct classification for each trajectory should be 5; (c) the relative entropy should be 0.5; and (d) the minimum number of individuals assigned to each trajectory should exceed 3% of the total population. Of the models that met all the above criteria, one final model was identified based on the clinical interpretability of the number and shape of the trajectories and the Bayesian information criterion. The eGFR trajectories were depicted using parametric polynomial functions obtained from the GBTM that smooth longitudinal patterns over time. Missing outcome data are handled within a maximum likelihood framework under the missing-at-random assumption, allowing individuals with incomplete follow-up to contribute to trajectory estimation. Scatter plots were constructed using the measured eGFR values.

For the second step, we investigated patient characteristics according to each trajectory and CKD stage. Continuous variables were summarized as means with standard deviations, and categorical variables were reported as frequencies and percentages. We calculated the eGFR slope for each trajectory and CKD stage using 2 methods: (1) using the prediction equation obtained from the GBTM, and (2) using the absolute change in measured eGFR (calculated as the final eGFR minus the initial eGFR).

For the third step, we evaluated the relationship between the eGFR trajectory as the dependent variable and patient characteristics as the independent variables using logistic regression analysis. In the multivariate analysis, we pre-specified 12 variables as candidate factors, comprising sex, age, body mass index, albumin and hemoglobin levels, presence of hypertension, diabetes, hyperlipidemia and hyperuricemia, presence of proteinuria and hematuria and treatment intervention, based on the previous study [17] and clinical perspectives. The results are reported as odds ratios, representing the risk of progressive eGFR decline with 95% confidence intervals.

All analyses were performed using the Stata software. Statistical significance was set at *p* < 0.05.

## Results

**Fig. 1:**
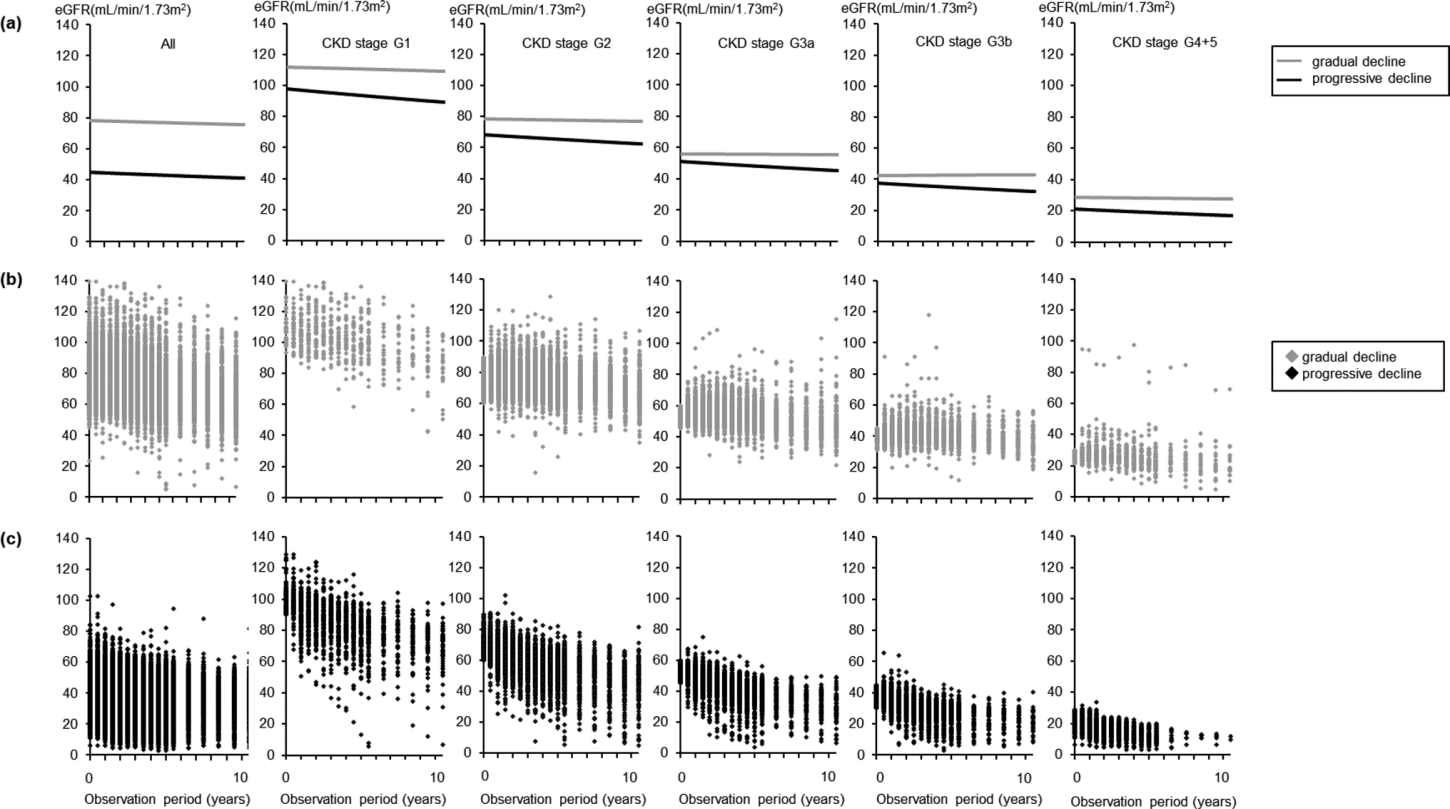
Trajectories and scatter plots of estimated glomerular filtration rate (eGFR) over 10 years. **a** 2 distinct trajectories with the progressive and gradual eGFR declines identified by group-based trajectory modeling; (**b**) Scatter plots of eGFR in the gradual decline; and (**c**) Scatter plots of eGFR in the progressive decline. We selected a model fitting a quadratic function to the 2 groups that met all the selection criteria and that was clinically interpretable. The gradual decline with gray lines or dots indicates an almost stable eGFR or maintained eGFR. The progressive decline with black lines or dots indicates a slightly steeper decline in the eGFR over time

Of the 2379 patients, 2257 had ≥ three eGFR measurements during the observation period. We selected a model fitting a quadratic function (nonlinear) to the 2 trajectory groups that met all selection criteria and was clinically interpretable because the three trajectory groups were not classified at G 4 + 5 (Online Resource Table 1). Two distinct trajectories of eGFR decline were identified: the progressive decline (*n* = 1240, 54.9%) with a baseline GFR of 44.8 ml/min/1.73 m^2^, and the gradual decline (*n* = 1017, 45.1%) with a baseline GFR of 78.3 ml/min/1.73 m^2^. Regarding the eGFR trajectories for each CKD stage, the proportions of progressive and gradual decline were 71.6 and 28.4% in G1, 48.9 and 51.1% in G2, 45.9 and 51.1% in G3a, 54.3 and 45.7% in G3b, and 62.8 and 37.1% in G4 + 5, respectively (Fig. 1 and Table 1).

**Table 1:** Baseline patients’ characteristics (*n* = 2257)

	All (*n* = 2257)		G1 (*n* = 191)		G2 (*n* = 858)		G3a (*n* = 598)		G3b (*n* = 400)		G4+5 (*n* = 210)	
	Trajectory group		Trajectory group		Trajectory group		Trajectory group		Trajectory group		Trajectory group	
Variables	progressive decline	gradual decline	progressive decline	gradual decline	progressive decline	gradual decline	progressive decline	gradual decline	progressive decline	gradual decline	progressive decline	gradual decline
Number (%)	1240 (54.9)	1017 (45.1)	137 (71.6)	54 (28.4)	420 (48.9)	438 (51.1)	275 (45.9)	323 (54.1)	217 (54.3)	183 (45.7)	132 (62.8)	78 (37.1)
Male sex, *n* (%)	871 (70.1)	752 (74.1)	90 (65.7)	40 (74.1)	311 (74.4)	330 (75.0)	212 (77.4)	248 (76.5)	148 (68.8)	131 (70.8)	78 (58.7)	35 (45.5)
Mean age (years)	64.9±7.6	60.8±8.7	58.7±8.2	55.6±8.2	63.0±8.2	60.3±8.8	65.1±7.5	63.7±7.9	65.7±6.9	65.4±7.2	64.6±8.1	66.6±7.5
Body mass index	25.6±3.8	25.9±4.0	26.4±4.1	26.1±4.3	25.7±3.7	26.0±4.0	25.6±3.6	25.8±4.3	25.3±3.5	25.4±3.9	25.5±3.8	25.7±3.3
Systolic blood pressure (mmHg)	136±14	137±13	136±14	138±12	138±12	136±12	136±13	135±14	138±15	134±12	141±15	134±14
Diastolic blood pressure (mmHg)	78±9	79±9	80±8	80±8	79±8	79±9	77±+9	78±10	78±10	78±9	77±10	77±9
Albumin (g/dL)	4.2±0.4	4.3±0.3	4.3±0.4	4.4±0.3	4.3±0.3	4.3±0.3	4.1±0.4	4.3±0.3	4.1±0.4	4.2±0.4	4.0±0.4	4.3±0.2
Hemoglobin (g/dL)	13.3±1.9	14.3±1.5	14.2±1.6	14.4±1.7	14.2±1.6	14.4±1.4	13.7±1.7	14.1±1.7	12.9±1.7	13.6±1.8	11.6±1.7	12.2±1.5
Hemoglobin A1c (%)	6.2±1.1	6.5±1.2	6.7±1.3	7.1±1.4	6.6±1.3	6.4±1.2	6.3±1.0	6.0±0.9	6.1±1.1	6.1±1.0	5.9±0.9	6.1±1.3
Total cholesterol (mg/dL)	195±35	199±36	202±33	201±38	201±37	198±36	196±32	195±33	197±36	191±36	192±43	198±34
Serum uric acid (mg/dL)	6.5±1.5	5.7±1.5	5.4±1.5	5.1±1.1	5.9±1.5	5.7±1.5	6.5±1.4	6.2±1.6	6.6±1.6	6.7±1.8	6.8±1.5	7.0±1.3
Comorbidities, *n* (%)												
Hypertension	1151 (93.1)	893 (88.2)	117 (85.4)	48 (88.9)	370 (88.5)	382 (87.2)	258 (94.5)	303 (93.8)	202 (94.4)	169 (92.0)	127 (96.2)	68 (88.3)
Diabetes	683 (55.2)	701 (69.1)	109 (79.6)	44 (81.5)	285 (68.2)	295 (67.2)	156 (57.1)	176 (54.5)	126 (58.9)	97 (52.7)	62 (47.0)	34 (44.2)
Hyperlipidemia	854 (69.0)	703 (69.4)	94 (68.6)	37 (68.5)	284 (67.9)	306 (69.9)	190 (69.6)	229 (70.9)	151 (70.6)	133 (72.3)	86 (65.2)	47 (61.0)
Hyperuricemia	632 (51.1)	245 (24.2)	21 (15.3)	10 (18.5)	126 (30.1)	97 (22.2)	124 (45.4)	138 (42.7)	124 (57.9)	103 (55.4)	90 (68.2)	45 (58.4)
Renal function												
Mean serum creatine (mg/dL)	1.34±0.55	0.76±0.16	0.60±0.09	0.55±0.09	0.84±0.13	0.75±0.12	1.11±0.18	1.01±0.14	1.43±0.25	1.27±0.22	2.52±0.72	1.74±0.38
Mean eGFR (mL/min/1.73m^2^)	44.4±13.7	77.7±15.4	96.8±8.9	111.9±17.1	67.4±7.3	77.6±8.3	50.0±5.5	55.6±5.2	37.0±5.4	42.4±6.4	20.5±5.0	28.6±8.8
Presence of proteinuria, *n* (%)	787 (71.9)	547 (61.3)	79 (64.2)	27 (57.5)	256 (68.3)	227 (59.1)	196 (79.0)	163 (58.0)	146 (79.4)	97 (59.2)	95 (86.4)	48 (67.6)
Presence of hematuria, *n* (%)	252 (24.0)	149 (17.2)	16 (13.6)	2 (4.4)	70 (19.2)	78 (21.1)	72 (30.5)	41 (15.1)	54 (30.5)	32 (20.0)	23 (21.5)	13 (18.8)
Intervention groups*, *n* (%)												
Standard intervention	602 (48.5)	540 (53.2)	72 (52.5)	31 (57.4)	222 (53.1)	242 (55.0)	136 (49.6)	159 (49.1)	90 (41.9)	86 (46.5)	70 (52.6)	34 (44.2)
Advanced intervention	640 (51.5)	475 (46.8)	65 (47.5)	23 (42.6)	196 (46.9)	198 (45.0)	138 (50.4)	165 (50.9)	125 (58.1)	99 (53.5)	63 (47.4)	43 (55.8)

Table 1 shows baseline patient characteristics for all CKD stages according to each trajectory. In the progressive eGFR decline group, 871 (70.1%) of the patients were male, the mean age was 64.9 years, serum creatinine was 1.34 mg/dL, and eGFR was 44.4 mL/min/m^2^. In the gradual eGFR decline group, 752 (74.1%) of the patients were male, the mean age was 60.8 years, serum creatinine was 0.76 mg/dL, and eGFR was 77.7 mL/min/m^2^.

Table 2 shows annual eGFR slope by two measurement methods. For all patients, the GFR slope calculated by predictive equation was −0.35 mL/min/1.73 m^2^/year in the progressive decline and −0.30 mL/min/1.73 m^2^/year in the gradual decline. The GFR slope calculated by measured values was −1.26 (−2.25, − 0.49) mL/min/1.73 m^2^/year in the progressive decline, and −1.25 (−2.46, −0.21) mL/min/1.73 m^2^/year in the gradual decline. At every CKD stage, the speed of progressive decline was faster than that of gradual decline.

**Table 2:** Annual eGFR slope during observation period by measurement methods

	All		G1		G2		G3a		G3b		G4+5	
	Trajectory group		Trajectory group		Trajectory group		Trajectory group		Trajectory group		Trajectory group	
	progressive decline	gradual decline	progressive decline	gradual decline	progressive decline	gradual decline	progressive decline	gradual decline	progressive decline	gradual decline	progressive decline	gradual decline
*n*	*n* = 1240	*n* = 1017	*n* = 137	*n* = 54	*n* = 420	*n* = 438	*n* = 275	*n* = 323	*n* = 217	*n* = 183	*n* = 132	*n* = 78
Baseline eGFR (mL/min/1.73m^2^)	44.8	78.3	98.1	112.0	68.5	78.2	51.4	56.2	37.8	42.2	21.2	28.6
eGFR slope calculated from the predictive equation	−0.35	−0.30	−0.88	−0.26	−0.62	−0.14	−0.60	−0.05	−0.55	0.05	−0.41	−0.12
*n*	n = 239	*n* = 281	*n* = 44	*n* = 15	*n* = 92	*n* = 133	*n* = 52	*n* = 90	*n* = 28	*n* = 49	*n* = 2	*n* = 15
Baseline eGFR (mL/min/1.73m^2^)	45.1	74.7	96.0	109.2	66.2	76.8	50.1	54.9	36.6	41.7	21.1	26.6
eGFR slope calculated from measured values ^a)^	−1.26 (−2.25, −0.49)	−1.25 (−2.46, −0.21)	−2.97 (−3.82, −2.10)	−2.53 (−4.53, −1.38)	−2.12 (−3.34, −1.23)	−0.79 (−1.59, −0.05)	−2.35 (−2.78, −1.71)	−0.74 (−1.17, 0.10)	−1.42 (−2.09, −0.77)	−0.32 (−1.15, 0.35)	−0.96 (−1.11, −0.82)	−0.50 (−0.87, 0.02)

eGFR, estimated glomerular filtration rate

^a^Values shown are median (25th, 75th percentile)

Table 3 shows the results of the logistic analysis of the patient characteristics in the progressive eGFR decline with gradual eGFR decline as the reference. Of the 17 variables, 13 (sex, age, diastolic blood pressure, lower albumin, hemoglobin, and hemoglobin A1c levels, higher serum uric acid level, hypertension, hyperuricemia, absence of diabetes, proteinuria, hematuria, and treatment intervention) were associated with progressive eGFR decline among all patients.

**Table 3:** Logistic regression analysis for patient characteristics associated with progressive eGFR decline

	All		G1		G2		G3a		G3b		G4+5	
Variables	OR	95% CI	OR	95% CI	OR	95% CI	OR	95% CI	OR	95% CI	OR	95% CI
Sex (male = 0, female =1)	**1.22**	1.01–1.47	1.49	0.74–3.02	1.03	0.76–1.40	1.00	0.68–1.47	1.10	0.72–1.69	0.59	0.33–1.03
Age	**1.06**	1.05–1.08	**1.05**	1.01–1.09	**1.04**	1.02–1.05	1.02	1.00–1.04	1.00	0.98–1.03	0.97	0.93–1.00
Body mass index	0.98	0.96–1.01	1.02	0.94–1.10	0.98	0.94–1.01	0.99	0.95–1.03	0.99	0.94–1.05	0.99	0.91–1.07
Systolic blood pressure (mmHg)	1.00	0.99–1.01	0.99	0.97–1.02	**1.01**	1.00–1.03	1.01	1.00–1.02	**1.02**	1.01–1.04	**1.03**	1.01–1.05
Diastolic blood pressure (mmHg)	**0.98**	0.97–0.99	0.99	0.95–1.03	1.00	0.98–1.01	0.99	0.98–1.01	1.00	0.98–1.02	1.01	0.98–1.04
Albumin (g/dL)	**0.21**	0.15–0.29	**0.24**	0.06–0.96	**0.48**	0.29–0.78	**0.23**	0.12–0.43	**0.28**	0.14–0.57	**0.07**	0.02–0.26
Hemoglobin (g/dL)	**0.71**	0.67–0.75	0.92	0.74–1.14	0.91	0.82–1.00	**0.83**	0.75–0.93	**0.81**	0.72–0.92	**0.78**	0.64–0.95
Hemoglobin A1c (%)	**0.78**	0.72–0.85	0.82	0.64–1.04	1.10	0.97–1.24	**1.28**	1.04–1.58	1.05	0.84–1.31	0.90	0.65–1.23
Total cholesterol (mg/dL)	1.00	0.99–1.00	1.00	0.99–1.01	1.00	1.00–1.01	1.00	1.00–1.01	1.00	1.00–1.01	1.00	0.99–1.00
Serum uric acid (mg/dL)	**1.48**	1.38–1.59	1.17	0.90–1.53	1.08	0.98–1.19	1.12	1.00–1.26	1.00	0.88–1.12	0.91	0.74–1.13
Comorbidities												
Hypertension	**1.80**	1.34–2.40	0.73	0.28–1.93	1.13	0.75–1.70	1.13	0.57–2.25	1.49	0.68–3.28	**3.36**	1.08–10.43
Diabetes	**0.55**	0.46–0.66	0.88	0.40–1.97	1.05	0.79–1.39	1.10	0.80–1.52	1.28	0.86–1.91	1.12	0.64–1.97
Hyperlipidemia	0.98	0.82–1.18	1.00	0.51–1.98	0.91	0.68–1.22	0.93	0.65–1.32	0.92	0.59–1.42	1.19	0.67–2.13
Hyperuricemia	**3.27**	2.73–3.93	0.80	0.35–1.83	**1.52**	1.11–2.06	1.23	0.89–1.70	1.11	0.74–1.65	1.52	0.85–2.73
Renal function												
Presence of proteinuria	**1.62**	1.34–1.96	1.33	0.67–2.64	**1.49**	1.10–2.00	**2.67**	1.81–3.93	**2.65**	1.65–4.26	**3.03**	1.45–6.34
Presence of hematuria	**1.52**	1.21–1.91	3.45	0.76–15.65	0.89	0.62–1.27	**2.49**	1.61–3.84	**1.76**	1.06–2.90	1.18	0.55–2.52
Intervention groups												
(Standard intervention = 0	**1.21**	1.02–1.43	1.22	0.64–2.30	1.08	0.82–1.41	0.99	0.72–1.37	1.21	0.81–1.79	0.71	0.40–1.25
Advanced intervention = 1)												

Values shown are odds ratios (ORs) and 95% confidence intervals (CIs), with gradual eGFR decline as the reference

The results are highlighted in bold when the 95% CI did not exceed 1.0 (the null value)

Table 4 shows the results of the logistic regression analysis adjusted for patient characteristics excluding systolic and diastolic blood pressures, and hemoglobin A1c, total cholesterol, and serum uric acid levels, considering multi-collinearity. Increasing age, presence of hyperuricemia, and presence of proteinuria were associated with a higher risk of progressive eGFR decline, whereas lower body mass index, lower albumin and hemoglobin levels, and absence of diabetes were associated with a lower risk of progressive eGFR decline in renal function, in all patients. Lower albumin levels in CKD stages G2, G3a, and G4 + 5 and lower hemoglobin levels in CKD stages G3a to G4 + 5 were associated with progressive eGFR decline. The presence of proteinuria was also associated with a progressive eGFR decline in patients with CKD stages G2–G4 + 5.

**Table 4:** Adjusted associations by logistic regression analysis for patient characteristics with progressive eGFR decline

	All		G1		G2		G3a		G3b		G4+5	
Variables	OR	95% CI	OR	95% CI	OR	95% CI	OR	95% CI	OR	95% CI	OR	95% CI
Sex (male = 0, female =1)	1.05	0.76–1.46	1.88	0.61–5.79	1.21	0.73–1.99	0.94	0.49–1.80	0.86	0.43–1.73	0.58	0.22–1.53
Age	**1.06**	1.04–1.07	**1.10**	1.03–1.18	**1.04**	1.02–1.06	1.03	1.00–1.06	1.00	0.96–1.04	0.93	0.87–1.00
Body mass index	**0.96**	0.93–0.99	1.03	0.91–1.16	0.97	0.93–1.02	1.00	0.94–1.06	1.02	0.95–1.10	0.99	0.87–1.14
Albumin (g/dL)	**0.28**	0.18–0.42	0.23	0.04–1.50	**0.53**	0.30–0.96	**0.35**	0.16–0.78	0.47	0.22–1.02	**0.05**	0.01–0.35
Hemoglobin (g/dL)	**0.78**	0.71–0.85	1.36	0.97–1.89	1.00	0.87–1.16	0.90	0.77–1.05	**0.81**	0.68–0.97	**0.60**	0.42–0.85
Comorbidities												
Hypertension	1.18	0.76–1.82	0.30	0.07–1.32	1.13	0.63–2.00	1.02	0.40–2.60	0.67	0.20–2.30	2.21	0.25–19.63
Diabetes	**0.53**	0.41–0.70	0.77	0.20–2.99	0.94	0.62–1.42	0.85	0.52–1.40	1.37	0.78–2.42	1.34	0.51–3.52
Hyperlipidemia	1.04	0.78–1.38	1.58	0.54–4.60	1.10	0.72–1.67	1.29	0.76–2.20	0.78	0.41–1.47	1.28	0.50–3.28
Hyperuricemia	**4.42**	3.33–5.88	1.50	0.42–5.33	**1.63**	1.05–2.52	1.58	0.95–2.63	1.07	0.59–1.95	2.06	0.78–5.47
Renal function												
Presence of proteinuria, *n* (%)	**1.51**	1.15–1.99	2.24	0.90–5.58	**1.67**	1.12–2.47	**2.86**	1.68–4.88	**2.41**	1.26–4.59	**3.71**	1.23–11.20
Presence of hematuria, *n* (%)	1.22	0.89–1.69	1.63	0.25–10.58	0.69	0.43–1.09	**2.79**	1.51–5.17	1.13	0.59–2.14	0.56	0.17–1.90
Intervention groups												
Standard intervention = 0	1.18	0.92–1.52	0.98	0.38–2.49	1.22	0.85–1.76	1.12	0.70–1.81	1.23	0.70–2.17	0.61	0.24–1.56
Advanced intervention = 1												

Values shown are odds ratios (ORs) and 95% confidence intervals (CIs), with gradual eGFR decline as the reference

Analyses were adjusted for patient demographic and clinical characteristics, laboratory data, and the presence of comorbidities, considering multi-collinearity

The results are highlighted in bold when the 95% CI did not exceed 1.0 (the null value)

## Discussion

We evaluated changes in kidney function over 10 years in 2257 patients with CKD treated by primary care physicians in the FROM-J10 study. We demonstrated two distinct trajectories of eGFR with progressive and gradual decline using the GBTM and identified that the presence of proteinuria and lower albumin and hemoglobin levels were associated with progressive eGFR decline.

The most important finding was the depiction of the natural course of kidney function in patients with CKD who were properly managed by primary care physicians. Among all patients, the GFR slopes calculated by the predictive equation were −0.35 mL/min/1.73 m^2^/year in the progressive decline and −0.30 mL/min/1.73 m^2^/year in the gradual decline, which were similar, suggesting that the patient grouping might be affected by the differences in baseline eGFR as in previous study by Xie et al. [11]. We consider this to be a technical limitation of the analysis using GBTM. Our slopes were much slower than the rate of −2.27 ± 3.97 mL/min/1.73 m^2^/year in the study by Imasawa et al. [8]. Our decline rate is very close to the rate of GFR decline over 10 years of −0.36 in mL/min/1.73 m^2^/year in the Japanese general population aged 40–70 years reported by Imai et al. [1]. Our patients with CKD stage G4–5, the stage just before dialysis, did not follow the rapid decline shown 2 years before the initiation of dialysis in the study by O’Hare et al. [9]. We believe that even though our patients had CKD, appropriate CKD management by primary care physicians in cooperation with nephrologists prevented a rapid decline in kidney function.

The second important finding was the identification of the patient characteristics associated with progressive eGFR decline. In CKD stage G2–G5, the presence of proteinuria was associated with progressive decline, which is consistent with previous studies showing that proteinuria is a predictor of CKD progression independent of eGFR [1, 5, 6, 18]. The use of renin–angiotensin–aldosterone system inhibitors slows the progression of CKD and reduces proteinuria [19]. We suggest the importance of regular evaluation of proteinuria as an indicator of treatment effectiveness and selection because the evaluation of proteinuria is simple and inexpensive [20]. Lower albumin and hemoglobin levels are important clinical factors associated with decreased kidney function, which is consistent with the results of previous studies [21, 22]. Because treatment by nephrologists after nephrology referral slows CKD progression, especially among patients with albumin levels less than 3.5 g/dL and hemoglobin levels less than 11.0 g/dL [23], we recommend including lower albumin and hemoglobin levels in the nephrology referral criteria in the CKD guidelines. Complications other than hyperuricemia in CKD stage G2 were not associated with a progressive decline. This result is inconsistent with the finding that a history of hypertension was more likely in the low eGFR trajectory in community-dwelling older Japanese adults [13]. We considered that the higher prevalence of hypertension in our patients (88.2–93.1%) compared to 31–68% in participants by Sho et al. [13] led to no difference between eGFR trajectories. Regarding diabetes, the presence of diabetes was associated with a gradual eGFR decline, which is consistent with the Japanese community-based population study [24]. We considered that patients in the early stage of diabetic nephropathy with glomerular hyper-filtration might be included in CKD stages G1 and G2, and those were grouped into the gradual eGFR decline group [25]. The advanced intervention was associated with a progressive eGFR decline in CKD stages G2 to G3b as shown in Table 4 although it was not statistically significant. We considered that advanced interventions such as the initiation of renin–angiotensin system inhibitors might cause a subacute decline in kidney function, leading to these patients being grouped into a progressive eGFR decline group [26]. Further studies are required to examine the associations with treatment interventions including drug information.

We used the strength of the GBTM and its robustness in handling unbalanced or incomplete longitudinal data [14, 15] to analyze all data, including missing values seen in the latter 5 years of the FROM-J10 study. We classified patients within the same CKD stage into progressive and gradual eGFR decline groups and visually presented the changes in kidney function. Our primary aim was to characterize heterogeneity in longitudinal eGFR trajectories and classify patients into clinically interpretable subgroups, whereas a mixed effects model is better suited for quantifying average slopes and covariate effects on continuous outcome. We believe that these findings cannot be derived using conventional methods or sophisticated modeling techniques such as a mixed effects model. However, the GBTM has disadvantages such as dependence on subjective judgment in selecting the number of groups and functional forms, and overfitting with excessive group partitioning [14, 15]. To overcome these disadvantages, we conducted careful model selection and validation based on appropriate criteria and clinical interpretability. Regarding the number of groups, we selected 2 groups to standardize the conditions across all stages because patients with CKD stage G4–5 were not classified into three groups. This might limit the reflection of the reality of trajectories of kidney function in CKD stages from G1 to G3b. Regarding the shapes, we selected the quadratic function because its constant term was statistically significant and it reflected the more natural course of kidney function among linear, quadratic, and cubic shapes that all meet the criteria shown in Online Resource Table 1.

This study has some limitations. First, we could not evaluate the history of cardiovascular disease because it was not included in the questionnaire. Second, we must be careful in interpreting results obtained from multivariate analysis. The associations identified by multivariate logistic regression analysis do not imply causality. Separate logistic regression analysis for each CKD stage without adjustment might result in inflated Type I error rates. Third, the FROM-J 10 study was conducted before the standard use of sodium–glucose co-transporter 2 inhibitors in CKD treatment; therefore, the trajectories of kidney function reflected the effects of CKD clinical guideline-based treatment [16]. Finally, the potential seasonal validation in eGFR values was not considered in this analysis.

In conclusion, we showed two distinct eGFR trajectories over 10 years and demonstrated that the decline in kidney function was very slow in patients with CKD who were adequately treated by primary care physicians. We suggest that patient characteristics identified as progressive eGFR decline, proteinuria, lower albuminemia, and hemoglobin levels should be managed appropriately in routine clinical practice.

## Supplementary Information

Below is the link to the electronic supplementary material.

Supplementary file1 (DOCX 19 KB)

## Data Availability

The data sets used in this analysis are available from the corresponding author upon reasonable request.
