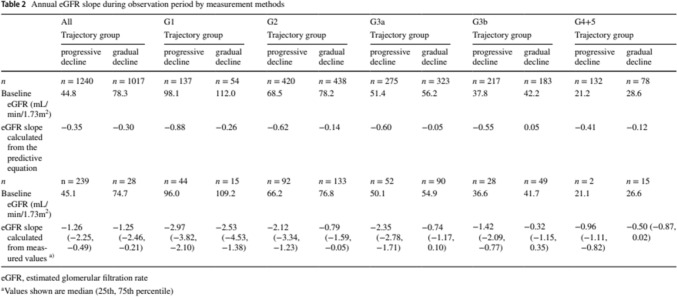# Correction: Trajectories of kidney function over 10 years in patients with chronic kidney disease: a 10 year follow-up of FROM-J study

**DOI:** 10.1007/s10157-026-02857-2

**Published:** 2026-04-13

**Authors:** Reiko Okubo, Masahide Kondo, Chie Saito, Hirayasu Kai, Ryoya Tsunoda, Akihiko Kato, Shoichi Maruyama, Jun Wada, Takashi Wada, Ichiei Narita, Kunihiro Yamagata

**Affiliations:** 1 Department of Nephrology, Institute of Medicine, University of Tsukuba, 1-1-1 Ten-Oudai, Tsukuba, Ibaraki Japan; 2 Department of Health Care Policy and Health Economics, Institute of Medicine, University of Tsukuba, Ibaraki, Japan; 3 Department of Clinical Laboratory Medicine, Institute of Medicine, University of Tsukuba, Ibaraki, Japan; 4 Ibaraki Clinical Education and Training Center, Institute of Medicine, University of Tsukuba, Tsukuba, Ibaraki Japan; 5 Department of Nephrology, Kosai Municipal Hospital, Kosai, Shizuoka Japan; 6 Department of Nephrology, Nagoya University Graduate School of Medicine, Nagoya, Japan; 7 Department of Nephrology, Rheumatology, Endocrinology and Metabolism, Okayama University Graduate School of Medicine, Dentistry and Pharmaceutical Sciences, Okayama, Japan; 8 Department of Nephrology and Rheumatology, Kanazawa University, Kanazawa, Japan; 9 Niigata Institute for Health and Sports Medicine, Niigata, Japan

**Correction: Clinical and Experimental Nephrology (2026) 30:632–642** 10.1007/s10157-026-02820-1

In Table 2 of this article, entries of value *n* = 28 in the [third] column were incorrect and should have been *n* = 281. The original article has been corrected and the corrected and incorrect version of Table 2 is shown below.

Corrected:

**Table 2:** Annual eGFR slope during observation period by measurement methods

	All		G1		G2		G3a		G3b		G4 + 5	
	Trajectory group		Trajectory group		Trajectory group		Trajectory group		Trajectory group		Trajectory group	
	Progressive decline	Gradual decline	Progressive decline	Gradual decline	Progressive decline	Gradual decline	Progressive decline	Gradual decline	Progressive decline	Gradual decline	Progressive decline	Gradual decline
*n*	*n* = 1240	*n* = 1017	*n* = 137	*n* = 54	*n* = 420	*n* = 438	*n* = 275	*n* = 323	*n* = 217	*n* = 183	*n* = 132	*n* = 78
Baseline eGFR (mL/min/1.73m^2^)	44.8	78.3	98.1	112.0	68.5	78.2	51.4	56.2	37.8	42.2	21.2	28.6
eGFR slope calculated from the predictive equation	− 0.35	− 0.30	− 0.88	− 0.26	− 0.62	− 0.14	− 0.60	− 0.05	− 0.55	0.05	− 0.41	− 0.12
*n*	*n* = 239	*n* = 281	*n* = 44	*n* = 15	*n* = 92	*n* = 133	*n* = 52	*n* = 90	*n* = 28	*n* = 49	*n* = 2	*n* = 15
Baseline eGFR (mL/min/1.73m^2^)	45.1	74.7	96.0	109.2	66.2	76.8	50.1	54.9	36.6	41.7	21.1	26.6
eGFR slope calculated from measured values^a^	− 1.26 (− 2.25, − 0.49)	− 1.25 (− 2.46, − 0.21)	− 2.97 (− 3.82, − 2.10)	− 2.53 (− 4.53, − 1.38)	− 2.12 (− 3.34, − 1.23)	− 0.79 (− 1.59, − 0.05)	− 2.35 (− 2.78, − 1.71)	− 0.74 (− 1.17, 0.10)	− 1.42 (− 2.09, − 0.77)	− 0.32 (− 1.15, 0.35)	− 0.96 (− 1.11, − 0.82)	− 0.50 (− 0.87, 0.02)

*eGFR* estimated glomerular filtration rate

^a^Values shown are median (25th, 75th percentile)

Incorrect: