# Supplementary material for: Trajectories of kidney function over 10 years in patients with chronic kidney disease: a 10 year follow-up of FROM-J study
Source: Clin Exp Nephrol. 2026 Jan 31;30(4):632–42. doi: 10.1007/s10157-026-02820-1 (PMC13009093; doi:10.1007/s10157-026-02820-1)
Supplement: Supplementary file 1 — Supplementary file1 (DOCX 19 KB) [file 10157_2026_2820_MOESM1_ESM.docx]

Trajectories of kidney function over 10 years in patients with chronic kidney disease: a 10-year follow-up of From-J study

Clinical and Experimental Nephrology

Reiko Okubo1,2,3, Masahide Kondo2, Chie Saito1, Hirayasu Kai1,4, Ryoya Tsunoda1, Akihiko Kato5, Shoichi Maruyama6, Jun Wada7, Takashi Wada8, Ichiei Narita9, Kunihiro Yamagata1

1 Department of Nephrology, Institute of Medicine, University of Tsukuba, Tsukuba, Ibaraki, Japan

2 Department of Health Care Policy and Health Economics, Institute of Medicine, University of Tsukuba, Ibaraki, Japan

3 Department of Clinical Laboratory Medicine, Institute of Medicine, University of Tsukuba, Ibaraki, Japan

4 Ibaraki Clinical Education and Training Center, Institute of Medicine, University of Tsukuba, Tsukuba, Ibaraki, Japan

5 Department of Nephrology, Kosai Municipal Hospital, Kosai, Shizuoka, Japan

6 Department of Nephrology, Nagoya University Graduate School of Medicine, Nagoya, Japan

7 Department of Nephrology, Rheumatology, Endocrinology and Metabolism, Okayama University Graduate School of Medicine, Dentistry and Pharmaceutical Sciences, Okayama, Japan

8 Department of Nephrology and Rheumatology, Kanazawa University, Kanazawa, Japan

9 Niigata Institute for Health and Sports Medicine, Niigata, Japan

Corresponding author: Kunihiro Yamagata

Department of Nephrology, Institute of Medicine, University of Tsukuba 1-1-1 Ten-oudai, Tsukuba, Ibaraki, Japan

E-mail: k-yamaga@md.tsukuba.ac.jp

Supplementary Table 1. Results of selection indicators for each model generated by group-based trajectory modeling

| Number | CKD stages | Shape | Bayesian information criterion | (a) Minimum average posterior probability of assignments | (b) Minimum odds of correct classification | (c) Relative  entropy | (d) Size of minimum group (%) | Judgment |
| --- | --- | --- | --- | --- | --- | --- | --- | --- |
| 2 groups | All | Linear | -105603.93 | 0.984 | 60.9 | 0.945 | 45.2 | Candidate |
|  |  | Quadratic | -105576.77 | 0.983 | 58.4 | 0.945 | 45.0 | Identified |
|  |  | Cubic | -105581.84 | 0.983 | 59.2 | 0.945 | 45.1 | Candidate |
|  | G1 | Linear | -9322.78 | 0.944 | 16.8 | 0.899 | 25.8 | Candidate |
|  |  | Quadratic | -9306.21 | 0.951 | 19.4 | 0.897 | 28.4 | Identified |
|  |  | Cubic | -9309.77 | 0.955 | 21.3 | 0.897 | 28.6 | Candidate |
|  | G2 | Linear | -37562.42 | 0.963 | 25.7 | 0.882 | 49.0 | Candidate |
|  |  | Quadratic | -37536.89 | 0.964 | 26.4 | 0.880 | 48.9 | Identified |
|  |  | Cubic | -37536.42 | 0.962 | 25.2 | 0.880 | 49.1 | Candidate |
|  | G3a | Linear | -24255.22 | 0.957 | 22.3 | 0.866 | 45.9 | Candidate |
|  |  | Quadratic | -24220.76 | 0.956 | 21.8 | 0.866 | 45.8 | Identified |
|  |  | Cubic | -24206.17 | 0.953 | 20.4 | 0.867 | 46.1 | Candidate |
|  | G3b | Linear | -15390.76 | 0.964 | 27.1 | 0.891 | 46.1 | Candidate |
|  |  | Quadratic | -15344.25 | 0.960 | 24.1 | 0.896 | 45.7 | Identified |
|  |  | Cubic | -15347.21 | 0.961 | 24.9 | 0.896 | 45.5 | Candidate |
|  | G4+5 | Linear | -6824.28 | 0.954 | 20.5 | 0.853 | 36.0 | Candidate |
|  |  | Quadratic | -6821.54 | 0.940 | 15.5 | 0.848 | 37.2 | Identified |
|  |  | Cubic | -6823.76 | 0.947 | 17.8 | 0.852 | 36.3 | Candidate |
|  |  |  |  |  |  |  |  |  |
| 3 groups | All | Linear | -100828.12 | 0.974 | 38.0 | 0.942 | 24.6 | Candidate |
|  |  | Quadratic | -100804.04 | 0.973 | 36.3 | 0.942 | 24.7 | Candidate |
|  |  | Cubic | -100809.38 | 0.973 | 35.9 | 0.942 | 24.7 | Candidate |
|  | G1 | Linear | -9101.74 | 0.926 | 12.5 | 0.914 | 7.3 | Candidate |
|  |  | Quadratic | -9094.39 | 0.939 | 15.3 | 0.907 | 10.3 | Candidate |
|  |  | Cubic | -9099.59 | 0.939 | 15.5 | 0.906 | 10.1 | Candidate |
|  | G2 | Linear | -36541.57 | 0.930 | 13.3 | 0.877 | 22.2 | Candidate |
|  |  | Quadratic | -36485.31 | 0.935 | 14.4 | 0.881 | 23.7 | Candidate |
|  |  | Cubic | -36486.28 | 0.936 | 14.6 | 0.881 | 23.6 | Candidate |
|  | G3a | Linear | -23576.11 | 0.937 | 14.8 | 0.869 | 24.6 | Candidate |
|  |  | Quadratic | -23528.89 | 0.932 | 13.7 | 0.874 | 24.1 | Candidate |
|  |  | Cubic | -23509.22 | 0.939 | 15.5 | 0.875 | 24.0 | Candidate |
|  | G3b | Linear | -15079.37 | 0.929 | 13.1 | 0.859 | 22.4 | Candidate |
|  |  | Quadratic | -15023.68 | 0.924 | 12.1 | 0.859 | 22.9 | Candidate |
|  |  | Cubic | -15025.45 | 0.928 | 12.9 | 0.862 | 22.5 | Candidate |
|  | G4+5 | Linear | -6370.74 | 0.964 | - | 0.928 | 0.48 | Violation |
|  |  | Quadratic | -6339.93 | 0.935 | - | 0.935 | 0.48 | Violation |
|  |  | Cubic | -6331.64 | 0.957 | - | 0.936 | 0.48 | Violation |

The appropriate model was determined on the basis of the following adequacy criteria: (a) the average posterior probability of assignments for each trajectory should be > 0.7, (b) the odds of correct classification for each trajectory should be > 5, (c) the relative entropy should be >0.5, and (d) the minimum number of individuals assigned to each trajectory should exceed 3% of the total population. Of the models that met all of the above criteria, one model was identified based on the Bayesian information criterion and clinical interpretability for the number and shape of the trajectories.　We selected a model fitting a quadratic function (nonlinear) to the two trajectory groups that met all selection criteria and was clinically interpretable in order to standardize the conditions across all stages, because the three trajectory groups were not classified at G 4+5.
